# Zebrafish as a Model for Epilepsy-Induced Cognitive Dysfunction: A Pharmacological, Biochemical and Behavioral Approach

**DOI:** 10.3389/fphar.2017.00515

**Published:** 2017-08-03

**Authors:** Uday P. Kundap, Yatinesh Kumari, Iekhsan Othman, Mohd. Farooq Shaikh

**Affiliations:** Neuropharmacology Research Laboratory, Jeffrey Cheah School of Medicine and Health Sciences, Monash University Malaysia Selangor, Malaysia

**Keywords:** zebrafish model development, epilepsy, anti-epileptic drugs, cognitive dysfunction, T-maze

## Abstract

Epilepsy is a neuronal disorder allied with distinct neurological and behavioral alterations characterized by recurrent spontaneous epileptic seizures. Impairment of the cognitive performances such as learning and memory is frequently observed in epileptic patients. Anti-epileptic drugs (AEDs) are efficient to the majority of patients. However, 30% of this population seems to be refractory to the drug treatment. These patients are not seizure-free and frequently they show impaired cognitive functions. Unfortunately, as a side effect, some AEDs could contribute to such impairment. The major problem associated with conducting studies on epilepsy-related cognitive function is the lack of easy, rapid, specific and sensitive *in vivo* testing models. However, by using a number of different techniques and parameters in the zebrafish, we can incorporate the unique feature of specific disorder to study the molecular and behavior basis of this disease. In the view of current literature, the goal of the study was to develop a zebrafish model of epilepsy induced cognitive dysfunction. In this study, the effect of AEDs on locomotor activity and seizure-like behavior was tested against the pentylenetetrazole (PTZ) induced seizures in zebrafish and epilepsy associated cognitive dysfunction was determined using T-maze test followed by neurotransmitter estimation and gene expression analysis. It was observed that all the AEDs significantly reversed PTZ induced seizure in zebrafish, but had a negative impact on cognitive functions of zebrafish. AEDs were found to modulate neurotransmitter levels, especially GABA, glutamate, and acetylcholine and gene expression in the drug treated zebrafish brains. Therefore, combination of behavioral, neurochemical and genenetic information, makes this model a useful tool for future research and discovery of newer and safer AEDs.

## Introduction

Epilepsy is a chronic neurological disorder characterized by unpredictable seizures, which may differ from a brief lapse of attention and muscle cramps to severe and long-lasting convulsions (Zashikhina, 2014). It has a poorly understood pathologic mechanism and is an intricate brain disorder with numerous fundamental causes (Galanopoulou et al., 2012). The multifactorial nature of epilepsy needs to be taken into consideration when developing therapeutic strategies overcoming selected mechanisms. Its clinical management is predominantly based on the administration of anti-epileptic drugs (AEDs) aiming to suppress the seizure activity. Although more than 20 AEDs are available, nearly 30% of the epileptic patients are refractory to drug-treatment. The AEDs are used to modify the processes involved in epileptogenesis and promote inhibition over excitation and thus prevent epileptic seizure (White et al., 2007). The AEDs act by several mechanism but the major mechanisms of action, include γ-aminobutyric acid (GABA) enhancers, glutamate blockers, calcium current inhibitors and sodium channel blockers (Czapinski et al., 2005). AEDs enhance inhibitory neurotransmission or suppress neuronal excitability. (Aldenkamp et al., 2003). Owing to the advantages of wide availability, lower cost and long-term experience older AEDs are still been prescribed, but greater effects are often exhibited by older AEDs (Eddy et al., 2011). Newer agents which are currently established have differences in their pharmacokinetic properties, mode of pharmacological action (Bootsma et al., 2009).

Cognitive impairment is a common comorbidity in multiple brain disorders like epilepsy, Alzheimer’s disease (AD), schizophrenia, Huntington’s disease (HD) and autism (Vingerhoets, 2006). Brain areas affected due to electric discharge in epilepsy are temporal lobe, hippocampus, medial frontal brain regions, bilateral superior temporal and subthalamus brain regions in epileptic patients (Laufs et al., 2007). It is better agrued that seizure-induced neuronal modeling during epilepsy and recurrent seizures can cause continuous neuronal reorganization (Pitkänen and Sutula, 2002). As temperol lobes and hippocampus is associated with memory formation, it is not shocking that epilepsy in such area can cause memory dysfunctioning (Helmstaedter and Kockelmann, 2006). Neurotransmitters are important in maintaining the normal brain functions and also known to be modulated during a brain insult. Alteration of neurotransmitters has been found to be closely associated with epilepsy. Some important neurotransmitters known to paly a significant role in epilepsy and cognition are GABA, glutamate and acetylcholine (Sancheti et al., 2013). GABA is an inhibitory transmitter and known for its role in suppressing epilepsy (Rico et al., 2011). Glutamate is an excitary chemical which causes neuronal death and is associated with glutamate neuro toxicity in epilepsy (Ozawa et al., 1998). Acetylcholine (ACh) plays the key role in modulating glutamate release and memory formation. It is reported that AEDs reduce neuronal irritability but also may impair neuronal excitability, neurotransmitter release, enzymes, genes and factors critical for information processing and memory (Hamed, 2009). As per a clinical study done at Columbia University, New York, NY, United States over all AEDs related cognitive side effect (CSEs) was found to be 12.8%. Drug specific CSEs for gabapentin (7.3%), oxcarbazepine (11.6%), phenytoin (12.9%). From the study, it was concluded that patient-reported CSEs are most common with topiramate (TPM), followed by zonisamide, phenytoin, and oxcarbazepine (Arif et al., 2009).

**FIGURE 1:**
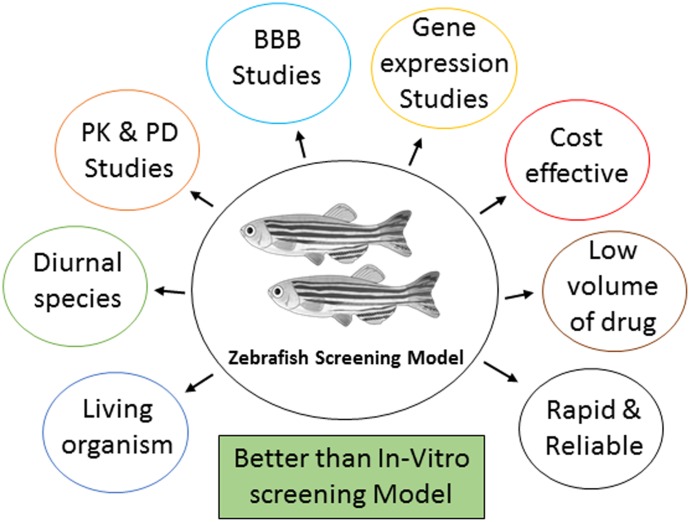
The significance of using zebrafish as a screening model.

Zebrafish has a complex nervous system capable of sophisticated behaviors and susceptible to seizures. A full range of mature behavior can be studied in adult zebrafish which makes them particularly desirable for model development. In last few years, the use of zebrafish has gained popularity as an alternative to rodents and other experimental animals for the study of the molecular mechanisms underlying cognition deficit and for the screening of potential therapeutic compounds (Kalueff et al., 2014). There is much scientific evidence illustrating the benefits of animals, such as zebrafish, as a replacement and better animal model for drug discovery (**Figure 1**). Genetic structure of zebrafish is similar to human. Around 70 percent of genes are shared with humans and about 84 percent of genes known to human disease are also expressed in zebrafish (Norton and Bally-Cuif, 2010). The overall cost of the studies which involves large animals are expensive and laborious. The small animal or rodent models are preferred to study safety profile of a drug and for testing of the hypothesis (Jucker, 2010). The non-human primate and rodents models are similar to humans in terms of their anatomy, physiology and behavior, but they are not so common due to ethical and economical concerns (Jucker, 2010). The cost and time required to carry out the study in zebrafish model is less as compared to rodents (Mussulini et al., 2013). For studying various brain disorders, zebrafish (*Danio rerio*) is quickly mounting as a promising model creature (Kundap et al., 2016). It is important to minimize animal distress by using the least sensitive organism possible to answer the question at hand. One of the biggest challenges in conducting research on epilepsy and cognitive function is the absence of a precise, sensitive, and reproducible disease model (Vining, 1987).

Pentylenetetrazole (PTZ) is used in zebrafish to induce epileptic seizure-like condition and to study the mechanisms of seizures (Desmond et al., 2012). γ-aminobutyric acid (GABA) is the major inhibitory neurotransmitter in the brain, PTZ exerts its convulsive effect by inhibiting the activity of GABA at GABA_A_ receptors (Shaikh et al., 2013). There are several reports which provide strong evidence on the usefulness of zebrafish model for studying cognitive functions. Investigators used active avoidance paradigm to discover a high impact about learning and memory in zebrafish. In this method zebrafish are trained to associate light with shock stimulus in a fish shuttle-box (Xu and Goetz, 2012). Zebrafish has natural color preference choice and it has received little attention in past few research. Natural color preference concerning a precise color may lead to changes in learning, decision making, memory and visual discrimination (Avdesh et al., 2010). The zebrafish is becoming an popular model increasingly for investigating treatment method and understanding the process behind memory problems in AD (Newman et al., 2014). The T-maze is a forthright method to test the learning skill, long- and short-term memory, and memory plasticity in zebrafish (Vignet et al., 2013). The T-maze has been most comprehensively used to examine specific features of spatial working and learning memory (Wenk, 2001).

In the view of current literature, the prime goal of the study was to develop a zebrafish model of epilepsy induced cognitive dysfunction and simulate the clinical condition which shows that both epilepsy and AEDs negatively affect the cognitive functions. This zebrafish model will serve as an important tool for the development and screening of newer and safer anti-epileptic drugs with intact memory functions. As epilepsy involve important biological and physiological mechanisms, studying zebrafish behavior, neurotransmitters level and gene expression are of great significance and key parameters in the development of an impressive animal model.

## Materials and Methods

### Chemicals and Equipment

Glutamic acid (Glu), γ-aminobutyric acid (GABA), Acetylcholine (ACh) and Pentylenetetrazole (PTZ) were from Sigma–Aldrich (United States). All the standard AEDs such as Phenytoin (PHY), Oxcarbazepin (OXC), Gabapentin (GBP), Diazepam (DZP), Rivastigmine (RSV) were from Sigma–Aldrich (United States). Dimethyl sulfoxide (DMSO) was purchased from Vivantis Inc (United States). Sony video recorder, Smart V3.0.05 tracking software (Pan Lab, Harvard apparatus), Agilent 1290 Infinity UHPLC, coupled with Agilent 6410 Triple, Quad LC/MS, Milli-Q system from Millipore (Bedford, MA, United States), Applied Biosystems StepOnePlus^TM^ Real-Time PCR Systems.

### Animal Care

**FIGURE 2:**
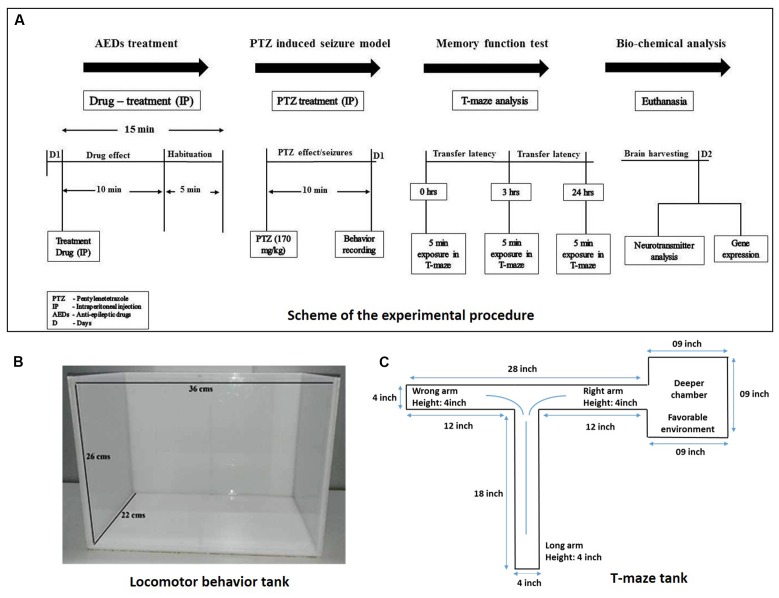
Experimental procedure and experiment tanks used. **(A)** Represents scheme for the experiment procedure used to carry out the study. **(B)** Represent the tank used for the locomotor behavior study. **(C)** Illustrates the typical size and specification of T-maze apparatus.

Adult zebrafish (*Danio rerio*; 3–4 months-old) of heterogeneous strain wild-type stock (standard short-fin phenotype) were obtained from a Akarium Batukarang, Subang Jaya, Malaysia. All fish were housed in the animal facility of Monash University Malaysia under standard husbandry conditions. Fish were maintained under temperature 28°C ± 2°C, pH between 6.8 and 7.1 and light intensity 250 lux with 14 h light: 10 h dark regime (light onset: 8am; light offset: 10pm). Fish were fed with TetraMin^®^ Tropical Flake and live Artemia from Bio-Marine Brand (Aquafauna, Inc. United States) three times a day to ensure a constant source of nourishment with *ad libitum* feeding. Standard zebrafish tank which are equipped with circulating water system with constant aeration having tank dimensions of (36 cm × 26 cm × 22 cm) were used as shown in **Figure 2**. Group housing (10–12 fishes/tank), males and females having seperate housing arrangement. All the animal experimentations were approved by Monash Animal Research Platform (MARP), Australia.

### Drug Treatment and Groups

Adult male zebrafish (*Danio rerio*) were used. PHY, OXC, GBP, DZP, RSV and PTZ were dissolved in 10% DMSO. Three months old fish were selected with a weight range of 0.5–0.6 g. Animal were divided into following groups, Group I: Vehicle control (10% DMSO); Group II: Pentylenetetrazole (PTZ-Negative control group); Group III: Phenytoin 80 mg/kg (PHY) + PTZ (170 mg/kg); Group IV: Oxcarbazepin 80 mg/kg (OXC) + PTZ (170 mg/kg); Group V: Gabapentin 800 mg/kg (GBP) + PTZ (170 mg/kg); Group VI: Diazepam 1.25 mg/kg (DZP) + PTZ (170 mg/kg); and Group VII: Rivastigmine 1.5 mg/kg (RSV) + PTZ (170 mg/kg). In the experiment 10–12 fisher group were used (**Figure 2**).

### Intraperitoneal Injection in Zebrafish

The vehicle, PTZ and AED treated groups were injected intraperitoneally (via posterior to the pelvic girdle into the abdominal cavity), using Hamilton syringe 10 μl (700 series, Hamilton 80400) (Stewart et al., 2011). The experiment was performed in a separate behavior room with constant room temperature of 28°C ± 2°C and humidity 50–60%. All the fish were acclimatized in the behavior room 2 h prior to experiment to avoid novel tank response. Precautions include using a small injection volume of 10 μl per gram of fish and a 35 gauge needle. Fish were restrained in water saturated sponge under benzocaine anesthesia to reduce the distress (Júnior et al., 2012). This technique of IP injection in zebrafish was found to be effective and did not cause any mortality throughout the experiment.

Fish was captured individually by fish holding net, then transfer into anesthesia solution (30 mg/L Benzocaine), Fish was taken out once anaesthesized and weighed to calculate the dose and the injection volume. A soft sponge of approximately 20 mm in height was saturated with water and set into 60 mm Petri dish. A cut of 10–15 mm deep was made on the sponge to restrain and hold the fish for injection. Intraperitoneal injection was made using a dissecting microscope by inserting the needle into the midline between the pelvic fins. Appropriate volume was injected according to the body weight. After injection, fish was immediately transferred to the tank.

### PTZ-Induced Seizure Model

The fish were treated with vehicle/AEDs via intraperitoneal injection and then habituated for 15 min in the observation tank before administration of PTZ. The vehicle control group only received 10% DMSO and suspended for behavior recording. Fifteen minutes after vehicle/AEDs administration, the animals were exposed to PTZ (170 mg/kg, IP) which presented different seizure profiles, intensities and latency to reach the scores. The seizures last for 10 min post-PTZ administration, which gradually decreases with time.. Adult zebrafish were tested in an observation tank, where the seizure score were measured using a special scoring system as mentioned in **Table 1**. Under the directives of Monash Animal Research Platform (MARP)-Australia, the dose of PTZ was adjusted to 170 mg/kg in order to get the highest seizure score of 4. Seizure score, seizure onset, total distance traveled, time spent in upper and the lower half of the tank were the parameters noted (Baraban et al., 2005).

**Table 1:** Seizure score analysis modified from Mussulini et al. (2013).

Seizure scores	Event of Behavior
Score 1	Short swim mainly at the bottom of the tank
Score 2	Increased swimming activity and high frequency of opercula movement.
Score 3	Burst swimming, left and right movements and erratic movements
Score 4	Circular movements

### T-maze Test

The T-maze is composed of one long (18′) and two short (12′) arms. One of the short arms is connected to a deeper square chamber (9 × 9′) which serve as a favorable environment for the fish (see **Figure 2**). Favorable environment is the chamber which is deeper and wider compare to other arms in T-maze and once fish finds it, they spend the majority of their time in it. The T-maze behavior test was performed in the behavior room of constant room temperature of 25°C – 26°C and humidity 50– 60%. Each fish was placed at the beginning of the long arm and the time required to reach deeper chamber was recorded in the 5 min exploration period. The time taken by the fish to travel into the deeper chamber was determined as transfer latency (TL). Transfer latencies were recorded at 0, 3, and 24 h post-PTZ exposure. The TL was expressed as inflection ratio (Kasture et al., 2007). Inflexion ratio (IR) = (L0-L1)/(L1), (IR) = (L0-L2)/(L2), where L0 is the initial latency (s) at 0 h and L1 and L2 is the latency (s) at 3 and 24 h trial. Behavior recording from seizure activity and T-maze test were analyzed for locomotor patterns. Tracking of the locomotor pattern was done by using computer software SMART v3.0-Panlab Harvard Apparatus^®^.

### Brain Harvesting

At the end of the behavioral studies, fish brains were harvested. The brain from each group was further divided into two sets and transferred into trizol for gene expression studies and methanol for LC-MS/MS respectively. The Brain was harvested by removing the skull of the fish and extracting the brain directly into the respective solvent. Brains were stored immediately at -80°C until further use.

### Neurotransmitter Analysis Using LC-MS/MS

Important neurotransmitters like GABA, glutamate, and acetylcholine (ACh) were analyzed using LC-MS/MS. Stock solutions of 1 mg/ml were prepared for all the standard neurotransmitters in methanol (0.1% formic acid). The solution was kept at 4°C until use.

Serial dilution from 100 to 2000 ppb was used for calibration. The brain was homogenized in 200 μl ice-cold methanol (1% formic acid). The homogenate was vortex-mixed for 1 min and then centrifuged at 18,000 × *g* for 10 min at 4°C. Finally, the supernatant was pipetted and placed into vials for LC-MS/MS analysis.

LC–MS/MS was run on an Agilent 1290 Infinity UHPLC, coupled with Agilent 6410 Triple Quad LC/MS, ZORBAX Eclipse plus C18 RRHD 2.1 × 150 mm, 1.8-micron (P/N 959759-902) column, the auto-sampler system (Agilent Technologies, Santa Clara, CA, United States). The samples were separated on a SMol-RRHD-Eclipse-C18-8 (15) UHPLC-160129-00011-Pos-DMRM used at 30°C. The mobile phase consisting of 0.1% formic acid in water (Solvent A) and acetonitrile with 0.1% formic acid (Solvent B) was used with a gradient elution: 0–3 min, 50% B; 3–6 min, 95% B; 06–07 min, 95% B at a flow rate of 0.1 ml/min. ESI-MS/MS Conditions were set as follows: ESI ion source, positive ion polarity, gas temperature 325°C, drying gas flow 9.0 L/min, nebulizer pressure 45 psi, Vcap 4000V. MS acquisition of GABA, Glu, ACh as performed in electrospray positive ionization multiple reaction monitoring (MRM) mode.

### Gene Expression

All brain samples were collected in ice-cold 200 μl TRIzol^®^ reagent (Invitrogen, Carlsbad, CA, United States) and stored at -80°C until use. Gene expression study was carried out for Neuropeptide Y (NPY), Brain-derived neurotrophic factor (BDNF) and cAMP-responsive element-binding protein1 (CREB1) genes.

### Isolation of RNA and First Strand cDNA Synthesis

Total mRNA were isolated by following the manufacturer’s protocol. In brief, brain tissues were appropriately homogenized in TRIzol^®^ reagent followed by mixing with chloroform and centrifuged at 13,500 rpm (revolutions per minute) for 15 min at 4°C. The upper aqueous supernatant was transferred into new tubes and isopropanol was added, mixed and were incubated for 10 min at room temperature and later centrifuged for 10 min at 13,500 rpm at 4°C. The supernatant was discarded and the pellets were subjected for rinsing with 75% ethanol. The pellets were then left for air drying between 5 and 8 min. Finally, nuclease-free water was added to each tube to dissolve the mRNA pellet. The concentration and purity of the isolated mRNA were measured by using NanoDrop Spectrophotometer. The mRNA samples were converted to cDNA with the help of Omniscript Reverse-transcription Kit (QIAGEN).

### StepOne^®^ Real-Time PCR

Gene expression for NPY, BDNF and CREB1 were computed using real-time quantitative RT-PCR (Applied Biosystems) using QuantiTect SYRB Green dye (Qiagen, Valencia, CA, United States). All the primer sets were purchased by Qiagen (npy: Dr_npy_1_SG QuantiTect Primer Assay (QT02205763), bdnf: Dr_bdnf_1_SG QuantiTect Primer Assay (QT02125326), creb:Dr_crebbpa_1_SG QuantiTect Primer Assay (QT02197503). Samples were incubated at 95°C for 2 min prior to thermal cycling (40 cycles of 95°C for 5 s and 60°C for 15 s). Relative expression of these three genes were attained by normalizing threshold cycle (Ct) values against Ct value of eef1a1b (housekeeping gene) (2 ^∧^ [Ct eef1a1b – Ct Gene of interest]).

### Statistical Analysis

All the data in the results are expressed as Mean ± Standard Errors of the Mean (SEM). Data is analyzed by Analysis of Variance (ANOVA) followed by Dunnett’s tests. The *P*-value ^∗^*P* < 0.05, ^∗∗^*P* < 0.01, and ^∗∗∗^*P* < 0.001 is considered as statistically significant. All the groups were compared with the PTZ negative control group.

## Results

### Seizure Onset Latency and Seizure Score Analysis

**FIGURE 3:**
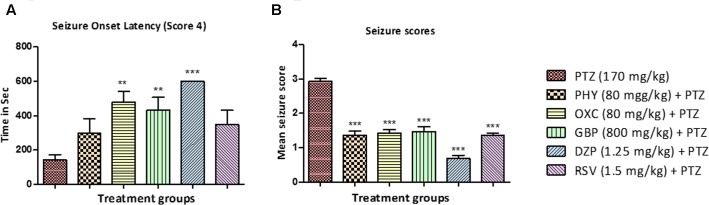
Seizure onset latency and behavior seizure score. **(A)** Represent seizure onset latency (score 4) of AEDs treated fish when compared with PTZ treated group. **(B)** Represent the effect of AEDs on PTZ induced seizure in zebrafish. Data are expressed as Mean ± SEM, *n* = 10 and statistical analysis by one-way ANOVA followed by Dunnett test. ^∗^*P* < 0.05, ^∗∗^*P* < 0.01, and ^∗∗∗^*P* < 0.001.

All the animals in the PTZ treated (Group II) reached seizure score 4 within 150 – 180 s after PTZ administration. In contrast, the onset was delayed in animals treated with standard AEDs as shown in **Figure 3A**. Drugs like, OXC, GBP, and DZP significantly delayed the onset of seizure whereas delay in onset with PHY and RSV was statistically insignificant. Seizures are measured using a special scoring system (**Table 1**). All the animals treated AEDs displays seizure not more than score 2 and exhibited seizure suppression activity. Thus a significant anti-epileptic activity was observed in all the animals treated with AEDs as shown in **Figures 3A,B** (^∗^*P* < 0.05, ^∗∗^*P* < 0.01, and ^∗∗∗^*P* < 0.001).

### Locomotor Pattern

**FIGURE 4:**
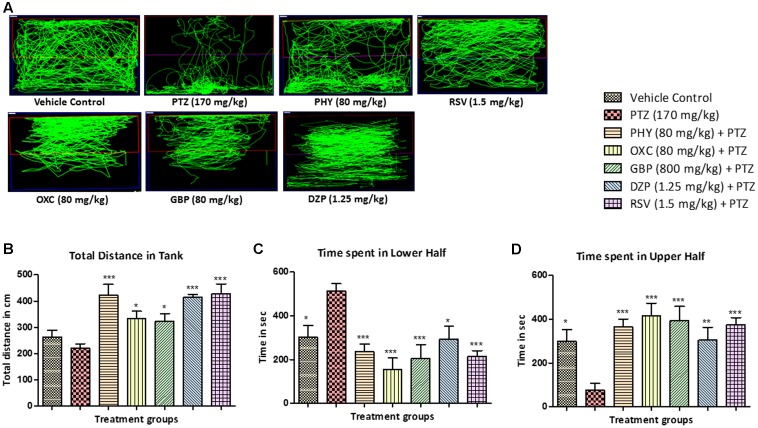
Pentylenetetrazole (PTZ) induced locomotor pattern and behavior. **(A)** Representative locomotion tracking patterns of vehicle control, PTZ treated and all the AEDs treated groups. **(B)** Represent the total distance traveled by the fish in experiment tank in different groups. **(C,D)** Represent the total time spent by each fish in the lower and upper half of the experimental tank. Data are expressed as Mean ± SEM, *n* = 10 and statistical analysis by one-way ANOVA followed by Dunnett test ^∗^*P* < 0.05, ^∗∗^*P* < 0.01, and ^∗∗∗^*P* < 0.001.

The locomotor pattern in the vehicle control group was demonstrated by normal swimming all over the tank. The PTZ group has spontaneously provoked seizures which is represented by abnormal and circular tracking pattern. The locomotor tracking pattern of PHY, RSV, OXC, GBP and DZP treated groups showed attenuation of PTZ seizure effect and a swimming pattern nearly similar to control group as shown in **Figure 4A**. The total distance traveled was significantly higher in all AED treated groups as compare to PTZ group. The total distance traveled was higher in control group as compare to PTZ group but it was found to be statistically insignificant as shown in **Figure 4B**. The control fish have spent the equal duration of time in both the halves of the tank, whereas PTZ group was found to be inconsistent in their swimming and spent more time in the lower half compared to the upper half of the tank. As fish were protected from seizure by treatment of AEDs, drug treatment reversed the PTZ seizure effect and time spent in lower half of the tank. So, AED treated group spent more time in upper half than compare to PTZ group as depicted in **Figures 4C,D** (^∗^*P* < 0.05, ^∗∗^*P* < 0.01, and ^∗∗∗^*P* < 0.001).

### Zebrafish T-maze Test for Anti-epileptic Drugs

**FIGURE 5:**
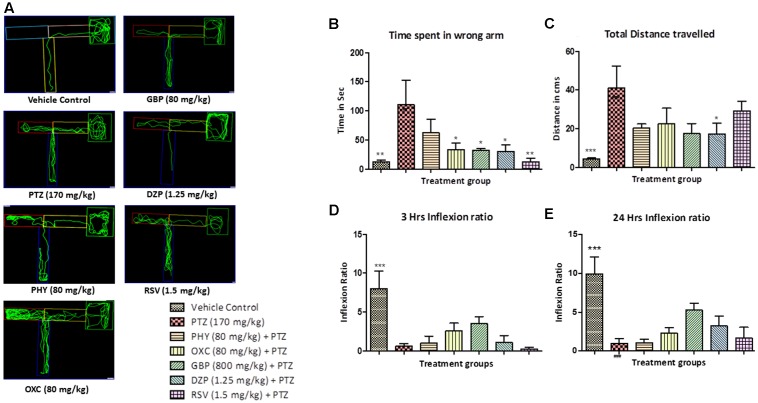
T-maze analysis and tracking pattern. In **(A)** each plot represents locomotor pattern of vehicle control, PTZ treated and all the AEDs treated groups. **(B,C)** represent total time spent in the wrong arm of T-maze by each fish and total distance traveled to reach the deeper chamber. **(D,E)** represents graph plot of inflecion ratio at 24 and 3 h T-maze trial. Data are expressed as Mean ± SEM, *n* = 10 and statistical analysis by one-way ANOVA followed by Dunnett test ^∗^*P* < 0.05, ^∗∗^*P* < 0.01, and ^∗∗∗^*P* < 0.001.

The control group showed less to no repeated entry into the wrong arm whereas PTZ group exhibited an opposite effect, so the time spent and total distance traveled was found to be significantly less as compared to PTZ treated group as shown in **Figures 5A,B**. T-maze tracking pattern of GBP and DZP shows single wrong entry in the T-maze. Whereas PHY, RSV and OXC treated group showed repeated entry to the wrong arm. Time spent in wrong arm by DZP, OXC, GBP and RSV was found to be significantly less as compared to PTZ group. PHY showed no significant reduction in time spend in wrong arm as shown in **Figure 5B**. AEDs treated group took the high/long time to reach the deepest chamber, and time spent in the wrong arm was also increased exhibiting impaired memory functions similar to PTZ group as shown in **Figure 5C**. Fish from vehicle control group exhibited an improved IR (memory function) at 3 and 24 h in the absence of seizures. PTZ treated group exhibited decreased IR both at 3 and 24 h. All the AEDs do not significantly increase IR (memory function) at 3 and 24 h when compared with PTZ group as shown in **Figures 5D,E**. In AED-treated groups, PTZ challenge had affected their IR, none of the AEDs significantly improved memory function when compared with PTZ group (^∗^*P* < 0.05, ^∗∗^*P* < 0.01, and ^∗∗∗^*P* < 0.001).

### Estimation of Neurotransmitters by LC/MS-MS

**FIGURE 6:**
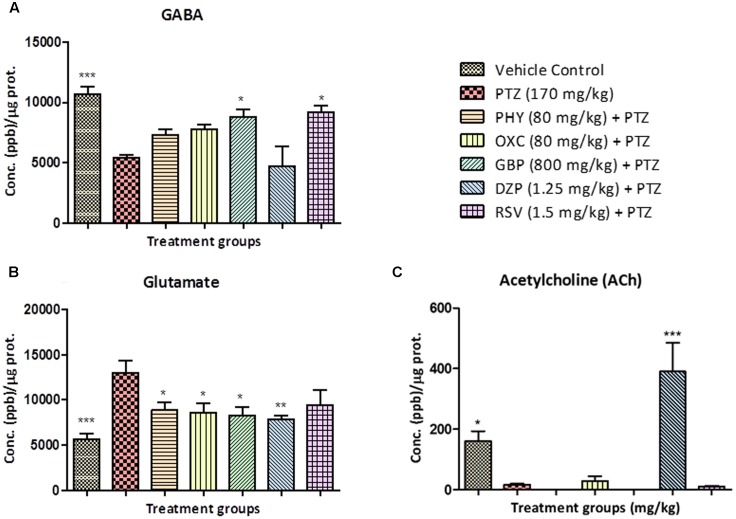
Concentration of neurotransmitters in zebrafish brain after T-maze test. Figure represents zebrafish neurotransmitter levels. **(A)** GABA, **(B)** Glutamate **(C)** Acetylcholine. Data are expressed as Mean ± SEM, *n* = 6 and statistical analysis by one-way ANOVA followed by Dunnett test ^∗^*P* < 0.05, ^∗∗^*P* < 0.01, and ^∗∗∗^*P* < 0.001.

Neurotransmitter analysis showed that, as PTZ is a GABA-_A_ receptor blocker the levels GABA in PTZ treated group were lower compared to control. No significant increase in GABA was found in PHY, OXC and DZP treated groups when compared to the PTZ group. GBP and RSV exhibited a significant increase in GABA as compared to the PTZ group as shown in **Figure 6A**. The level of glutamate in PTZ treated group was higher as compared to control group. Fish treated with AEDs significantly protected against PTZ induced glutamate surge and maintained glutamate levels similar to control group as shown in **Figure 6B**. Brain acetylcholine levels were significantly decreased by the PTZ group as compared with control. All the AEDs except DZP showed a similar ACh level as of PTZ group. DZP was the only drug that exhibited increased ACh levels as shown in **Figure 6C** (^∗^*P* < 0.05, ^∗∗^*P* < 0.01, and ^∗∗∗^*P* < 0.001).

### Estimation of Gene Expression by RT-PCR

**FIGURE 7:**
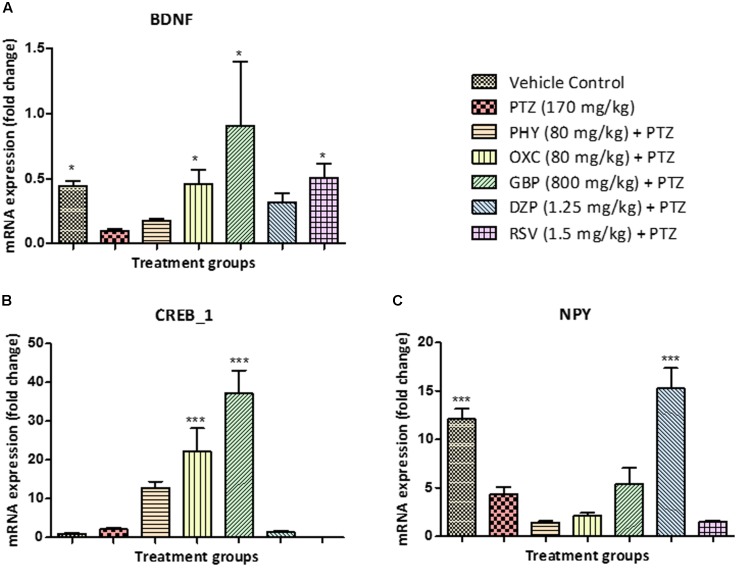
Gene expression in zebrafish brain determined by real time-PCR. **(A)** represents graph plot for BDNF mRNA expression in zebrafish brain, the level of BDNF fold change was found to be high in AEDs treated and control group as compared to PTZ treated group. **(B)** represents graph plot of CREB_1 mRNA expression levels in zebrafish brain, the level of CREB_1 fold change were found to be high in AEDs treated as compared to PTZ treated group. **(C)** represents graph plot of NPY mRNA expression in zebrafish brain, the level of NPY fold change were found to be high in DZP treated and control group as compared to PTZ treated group. This result shows that AEDs alters the gene expression fold change in the brain and affect the cognitive functioning of the brain. Data are expressed as Mean ± SEM, *n* = 6 and ^∗^*P* < 0.05, ^∗∗^*P* < 0.01, and ^∗∗∗^*P* < 0.001.

Brain-derived neurotrophic factor mRNA levels were downregulated in the PTZ treated group when compared with control. BDNF mRNA expression in PHY and DZP was found to be statistically insignificant. However, OXC, GBP, and RSV significantly upregulated mRNA expression of BDNF, as compared to PTZ treated group as shown in **Figure 7A**. CREB1 mRNA expression was up-regulated in the PTZ treated group as compared to the vehicle control group. In all AEDs treated fish except DZP and RSV, the mRNA expression of CREB_1 was similar as that of PTZ group, however, PHY, OXC, and GBP significantly upregulated the expression as shown in **Figure 7B**. NPY mRNA expression was downregulated in the PTZ treated group. However, this down-regulation was ameliorated by DZP pre-treatment as compared with PTZ group. This effect was same as observed in the control group. There was no significant difference observed in PHY, OXC and RSV pre-treatment when compared with PTZ treated group as shown in **Figure 7C** (^∗^*P* < 0.05, ^∗∗^*P* < 0.01, and ^∗∗∗^*P* < 0.001).

## Discussion

This work present development of a new model for epilepsy-induced cognitive dysfunction in zebrafish. This model has shown the effect of epilepsy on behavior, locomotion, important neurotransmitters and related genes expression. A considerable modulation in the parameters related to cognition such as learning and memory, proteins and genes were observed after epilepsy seizures. Effect of different AEDs’ was evaluated using the developed model. Findings of the this study suggests that AEDs significantly reverse the epileptic conditions but have some negative impact on the cognitive functions of the zebrafish.

For studying various brain disorders, zebrafish (*Danio rerio*) are gaining importance and emerging as a promising model organism (Guo et al., 2015). Seizure-like behavioral responses can be induced in adult zebrafish by several pharmacological approaches and there is an increasing trend of utilizing zebrafish model in epilepsy research (Kalueff et al., 2014). They are smaller in size and easy to maintain. Furthermore, it is a diurnal species with several fundamental similarities to humans (Stewart et al., 2014). As there is a limited spectrum of antiepileptic drugs (AEDs) available. A lot of patients are resistant to the existing therapy and many suffer from drug related comorbidities. In order to develop safe and efficient AEDs, there is a lack of model systems that fully recapitulate the condition of epilepsy induced cognitive dysfunction (Phillips and Westerfield, 2014). Zebrafish as an alternative to existing animal models is gaining popularity in the field of cognitive research. There are several reports which provide strong evidence on the usefulness of zebrafish model for studying cognitive functions (Stewart and Kalueff, 2012).

Pentylenetetrazole is a chemoconvulsant act via GABA_A_ receptor and act on an allosteric site (Desmond et al., 2012). A dose of 220 mg/kg of PTZ produce clonic tonic seizure in adult zebrafish (Banote et al., 2013). In the present study, a dose of 170 mg/kg was used as ethics committee suggested to use a lower dose to prevent further distress. Mussulini et al., 2013, utilized PTZ exposure to fish by dissolving pro-convulsant in the tank water (Mussulini et al., 2013). Whereas, IP injection in adult zebrafish was introduced by Kinkel et al. (2010). One of the method for administration of test substances to zebrafish is by dissolving the drug or chemical into the tank water, and it expected that test substance will be taken up by the fish. Nonetheless, tank water method do not give any idea about how much of the drug is actually absorbed or taken up by the fish (Kinkel et al., 2010). Intraperitoneal injection is considered as a better option to deliver a definite amount of drug to each fish, based on body weight calculations (10 μl for 1 gm fish). This route is of greater significance in order to correlate drug concentration to the efficacy and also important in metabolic studies (Traver et al., 2003). Intraperitoneal injection is relatively safe with zero mortality when tested in our laboratory. Hence, IP route of drug administration was preferred in our research.

In the present study, we have used seizsure score as described by Mussulini et al. (2013) with minor modifications. All the AEDs tested, delayed the onset of seizure in zebrafish after PTZ insult. This indicates the positive role of AEDs in preventing the seizure episode in patients (Patsalos et al., 2008). Latency to seizure score 4 is the time required by the fish to reach seizure score 4 in a given time frame of 600 s. Drugs like PHY, OXC, GBP, DZP and RSV significantly reduced seizure score when compared to PTZ treated group. The animals in the PTZ treated (Group II, negative control) exhibited seizure-like activity and full blown seizures of score 4 as shown in **Figures 3A,B**. Seizure scores mimic the clinical condition in the patients (Huang et al., 2001). Higher the score, more intense is the seizure episode. All the AEDs significantly helped in reducing the severity of the seizures as indicated by seizure score less than 2. It is also depicted by locomotor pattern as described earlier. Locomotor pattern clearly explains the abnormal behavior in PTZ group. The behavioral analysis and locomotor tracking patterns explain that the AEDs helped fish to overcome the PTZ effect and restored usual swimming movements nearly all over the tank. As seen in PTZ treated group the locomotor moment of the fish was observed to be at the bottom most part of the tank and time spent by fish was also more, which is similar to clinical stupor like behavior and anxiety in epileptic condition.

Seizures are the uncontrolled firing of neurons which produces involuntary moment of the body, which could be a partial or generalized type of seizures (Berg et al., 2010). Since greater the seizure frequency-duration and severity, it is likely to increase cognitive impairment in patients (Inoyama and Meador, 2015). Epidemiological studies reveal that dementia or memory problems is largely a unseen problem and the number is increasing (Farooq et al., 2007). Cognition in epilepsy can adversely be affected by multiple factors, including the seizure etiology, hereditary factors, psychosocial factors, and sequelae of epilepsy treatment, including AEDs (Motamedi and Meador, 2003). AEDs, primarily affects psychomotor speed, vigilance, and attention. Secondarily, it disturbs cognitive functions like memory and learning abilities (Chung et al., 2007). Children with age below 12 have a developing nervous system which is more susceptible to the long-standing side effects of AEDs-induced cognitive impairment. It is a serious problem and it is imperative to recognize and strategies to subside the negative effect of AEDs on cognition (Lagae, 2006). In a similar way, pharmacodynamic and pharmacokinetic factors both in combination are responsible for the cognitive effects of AEDs in individuals. For example, in the clinical setting, levetiracetam (LEV) and carbamazepine (CBZ) has reported adverse pharmacodynamic interactions (Sisodiya et al., 2002). LEV, TPM, CZB and other AEDs exhibit toxicity during combination therapy having a pharmacodynamic interaction (Perucca, 2006). Switching to another drug may help in preventing the damage and in improving cognitive function, memory, alertness, or ability to concentrate. Reports also shown that shifting from multi-drug therapy to monotherapy has been advantageous in reducing cognitive adverse effects (Vining et al., 1987; Wenk, 2001).

T-maze is used as an assessment tool which provides the brief overview of the cognitive status in a zebrafish model. T-maze is one of the most widely used behavioral paradigm to study detailed features of spatial working memory (Wenk, 2001). In this repetitive learning task, individual zebrafish were trained to explore and travel to the deeper end of the T-maze. Correct choices were rewarded with big space and favorable environment and incorrect choices result in getting small congested environment confinement (Lamb et al., 2012). In the present study, AEDs have significantly ameliorated seizure provoked by PTZ exposure but they have a negative impact on cognitive functions. Results from the T-maze test clearly demonstrated that AED-treated fish gets repeatedly lost in the T-maze, which shows epilepsy-related impaired spatial memory function in zebrafish. It is also observed that zebrafish have the capabilities to successfully learn, navigate and discriminate between wrong and right arm to reach the deeper chamber. Drugs like OXC, GBP and DZP have less negative impact than PHY and RSV on memory functions. The T-maze locomotor pattern seen in PTZ group was found to be significantly different as compared to control group. Most of the AEDs exhibit a similar locomotor pattern as of PTZ treated group in T-maze. The telencephalon connected to the olfactory organ is responsible for governing spatial memory, reproductive behavior, feeding behavior, and color vision in zebrafish. The spatial memory is accountable for recording information of individuals’s environment. A fish with poor working spatial memory may result in, getting repeatedly lost in the maze (Yu et al., 2006). The alteration in the neurotransmitter by AEDs and by epilepsy contributes to cognitive dysfunction in epilepsy patients (Greengard, 2001). There is a greater evidence available which explains the negative impact of AEDs on different aspects of cognitive functions.

Neurotransmitters are chemical agents in the body that modulate, initiate, and amplify signals across the brain (Thippeswamy et al., 2011). Abnormal alteration of neurotransmitters levels has been found to be closely associated with many neurological diseases including epilepsy (Huguenard, 2003). One of the reasons for the epileptic seizure and cognition in clinical conditions is neurotransmitter modulations by both epilepsy and AEDs (Park and Kwon, 2008; Kleen et al., 2010). LC–MS/MS method was used to quantify the neurotransmitters simultaneously in zebrafish brain (Santos-Fandila et al., 2015). It is well studied in rodents that neurotransmitters plays a significant role in modulating memory and learning function (Levin and Cerutti, 2009). A similar modulation was found in zebrafish brain when tested after T-maze test for learning and memory. GABA is an inhibitory transmitter in the central nervous system and known for its role in epilepsy (Shaikh et al., 2013). In the current research, all the fish treated with PTZ shows decrease level of GABA that contribute to epileptogenesis. The level of GABA was found to be high in the control group and some of the AEDs like DZP and GBP which acts via a GABAergic pathway. Drugs such as PHY and OXC which do not act via this pathway showed reduced GABA levels. A study performed by Silva (2003) in rodent, proves the fundamental role of GABA in controlling epilepsy (Silva, 2003; Werner and Covenas, 2011). The Increase in glutamate (Glu), an excitatory amino acid, is closely related to the etiology of epilepsy. This glutamate release causes a cascade of changes resulting in increased intracellular calcium that ultimately results in cell death (Holmes, 2002). It was found that level of glutamic acid in zebrafish brain of the control group was low as compared to PTZ treated group. In most of the AEDs treated group the level of glutamate was found to be high, which might have contributed to the neuronal loss, and impaired the memory function. By modulation of glutamate signaling, many biological events are related to brain functioning are affected (Ozawa et al., 1998), such as learning and memory (Izquierdo and Medina, 1997). Acetylcholine (ACh), a signaling molecule elicit several actions at neuromuscular junctions and in the CNS and also plays a key role in modulating glutamate release and maintaining memory formation. (Atzori et al., 2003). It was found that level of ACh in PTZ and all the AEDs treated group was low as compared to control group and this is in correlation with high levels of glutamate. Memory function was not improved due to the low level of ACh release in most of the AEDs treated group. But ACh was found to be high in DZP group and which could be due to acetylcholinesterase (AChe) inhibition property of DZP (Lundgren et al., 1987). Although the level of ACh in DZP treated group was high, there was not much improvement in memory functions in zebrafish when tested in T-maze.

Brain-derived neurotrophic factor, a member of the “neurotrophin” family, enhances the survival and differentiation of several classes of neurons (Mercer et al., 1997). In our result, we found that level of BDNF mRNA expression (fold change) in PTZ treated group was decreased. Similarly, we found that there was no significant difference in BDNF mRNA expression (fold change) in PHY and DZP treated groups, which showed that AEDs contributes toward memory impairment although correcting epilepsy. Fish treated with OXC, GBP, and RSV showed a slight increase in mRNA expression of BDNF gene. A study conducted by Binder (2004), have shown that upregulation of BDNF is involved in epileptogenesis, and modulation of BDNF signal transduction helps in preventing an epileptic condition (Binder, 2004). The transcription factor, CREB is crucial for important functions of cognition like memory and synaptic plasticity. Earlier studies reported an increase in CREB phosphorylation in rodent models of epilepsy (Zhu et al., 2012). In all the fish treated with AEDs, the level of CREB mRNA expression (fold change) was higher than PTZ treated group. This states that AEDs along with epilepsy contributes to loss of memory function in zebrafish after 24 h T-maze trial. The level of CREB mRNA expression was high in PTZ treated fish which shows that CREB too plays the important role in the process of epileptogenesis. Theories around cAMP-response element binding protein (CREB) and memory are still developing. Formation of long lasting memory is completely dependent on activation of (CREB)-dependent gene expression which is a crucial phase in the molecular pathway. Lower the expression of CREB gene higher will be the memory function (Benito and Barco, 2010). NPY play a significant role in regulating various physiological events in the brain, including energy balance, learning and memory, and epilepsy. NPY gene is generally responsible for regulation of neurotransmitters in the brain. It is reported that NPY gene therapy decreases chronic spontaneous seizures in a rat model of temporal lobe epilepsy (Noe et al., 2008). It selectively reduced synaptic excitation mediated by glutamate release (Hollopeter et al., 1998). In the zebrafish brain, neurons containing NPY mRNA are widely distributed in particular to regions like telencephalon, optic tectum, and rhombencephalon (Soderberg et al., 2000). In our result we found that level of NPY mRNA expression (fold change) in PTZ treated group was decreased. The mRNA expression of NPY gene in control group was found to be high. Similarly, fish treated with DZP and GBP, showed an increase in the mRNA expression of NPY gene. We found that there was no significant difference in NPY mRNA expression (fold change) in PHY, OXC, and RSV-treated group as compared with PTZ group. A study performed by Stroud et al., demonstrated that NPY suppresses absence seizures in Genetic Absence Epilepsy Rats of Strasbourg (GAERS) (Stroud et al., 2005). NPY also play a significant role in human epilepsy and it is supported by an increased NPY expression in biopsy samples from temporal lobe epilepsy patients (Furtinger et al., 2001).

This study suggest that the negative impact of standard anti-epileptic drugs on learning and memory, which is very common among epileptic patients. The successful development of zebrafish model was confirmed by the effect of epilepsy and AEDs impairing the learning and memory abilities in zebrafish. It was found that AEDs suppresses seizure-like behavior but cannot reverse seizure associated learning and memory dysfunction in a zebrafish model. This model, therefore, potentially extend the significant use of zebrafish and technique for screening or developing newer drugs for epilepsy-related cognition dysfunction in humans.

## Conclusion

For investigating the cause and pathology of human disease animal models are considered as a useful tool. It is better known that such models can never represent the complete pathology that is observed in human diseases. To develop a model in the animal for brain disorder particularly epilepsy is very difficult because of its disease complexity and variation among the species. However, by using a number of different techniques and parameters in the zebrafish, we can incorporate the unique feature of specific disorder to study the molecular and behavior basis of this disease. The behavioral study, neurotransmitter analysis and gene expression studies in the aforementioned work above provide a first proof-of-principle model for screening basic drugs in epilepsy-related cognitive research using zebrafish as a choice of animal.

## Ethics Statement

The experimental protocol was approved by the Monash Animal Research Platform (MARP) Animal Ethics Committee, Monash University, Australia (MARP/2016/009).

## Author Contributions

UK performed all the experimental procedure along with result analysis, manuscript writing and figures designing. YK contributed in designing gene expression study, result analysis and figures in the manuscript. IO contributed in LC-MS/MS method. MS contributed in designing the study, result analysis and manuscript writing.

## Conflict of Interest Statement

The authors declare that the research was conducted in the absence of any commercial or financial relationships that could be construed as a potential conflict of interest.
